# Motivation and Performance of Community Health Workers: Nothing New Under the Sun, and Yet…

**DOI:** 10.9745/GHSP-D-21-00627

**Published:** 2021-12-31

**Authors:** Eric Sarriot, Tom Davis, Melanie Morrow, Telesphore Kabore, Henry Perry

**Affiliations:** a Gavi, The Vaccine Alliance, Geneva, Switzerland.; b World Vision International, Geneva, Switzerland.; c ICF, Rockville, MD, USA.; d Save the Children, Washington, DC, USA.; e Johns Hopkins Bloomberg School of Public Health, Baltimore, MD, USA.

## Abstract

We know that both financial and nonfinancial incentives matter if we want community health workers (CHWs) who are motivated and performing. What are the practical implications for CHWs themselves and for effective management of viable CHW programs?

## Key Messages

- We consider the types of incentives that motivate community health workers' (CHWs) performance and the implications at 2 levels of systems analysis—first, for CHWs themselves and then for appropriate management of an invaluable human resource by CHW programs.
- The World Health Organization recommendations on commensurability of CHW compensation with the job demands—and corresponding professionalization—represent the culmination of decades of work, advances, forgetfulness, rediscovery, collective wisdom, and available evidence.
- Tapping into communities' own organizing potential through volunteerism is, however, a force in both high-income and low- and middle-income countries.

We offer predictions for the future direction of CHW programs:

- National CHW programs—whether implemented by states or non-state partners—will be a growing opportunity to viably strengthen national health systems.
- Partnerships between state and non-state actors will be key to sustain effective CHW programs in national PHC strategies at scale.
- The social-versus-institutional anchoring of CHW programs will be an enduring challenge for their performance and sustainability. Dual models (paid CHWs and volunteers), if ethically managed, will be a promising approach for efficiency and scale.

See related article by Roy et al.

## INTRODUCTION

*GHSP* has shared with some regularity research and practice papers on the recruitment, training, compensation, roles, support, potential, and performance of community health workers (CHWs). We are not alone in wanting CHW cadres to be taken seriously and engaged effectively. The (redundantly named) “community health community” took very favorably the recommendations of the World Health Organization (WHO) on CHW programs,^1^^,^^2^ which was the culmination of decades of work, advances, forgetfulness, rediscovery, and new lessons. The WHO recommendations provided an authoritative treatment of our collective wisdom and available evidence.^3^ In this issue of *GHSP*, Roy et al. bring us to Bangladesh—a country with notable governmental and nongovernmental efforts to structure CHW programs for effective primary health care (PHC)—to consider what factors drive the motivation, and consequently the performance, of CHWs.^4^ Roy et al. apply a robust qualitative methodology, provide insightful analyses, and offer a rich discussion about implications for program management. One of the findings is that^4^:

*CHWs shared a range of nonmonetary and monetary factors that have affected their motivation, performance, and job satisfaction.*

A cursory reader well-versed in CHW programs (for example, the 20-year-old seminal publication of Bhattacharyya et al. on CHW incentives and disincentives^5^) might be forgiven for pondering, “We already know that both financial and nonfinancial incentives matter if we want motivated and performing CHWs. What is new in this?” The question is timely as a new *Health Research Policy and Systems*^6^ supplement issue (including a summation of lessons on incentives and remuneration of CHWs^7^) now updates the work of Bhattacharyya et al.,^5^ the CHW Reference Guide,^8^ and more recently, the 29 country case studies by Perry on national CHW programs.^9^ Indeed, some findings recur across contextual studies and accrue a recognizable body of evidence, even if they do not have the sharp edges of controlled experiments.

As we are in the age of “systems thinking” in global health, we consider 2 systems of a different order: the biological-psychosocial system of CHW workers as individuals and the social-institutional system in which CHW programs are anchored.

## PEOPLE ARE SYSTEMS TOO

We can examine some of the ways to break down the incentives and disincentives to CHW motivation, starting with the central question (for programmers) of financial compensation and the thorny corollary issue of volunteerism.

Without going out on a limb, we observe that throughout history, regardless of the field of practice, financial compensation has been a consistent incentive for giving one's time and energy to a project or corporate entity. Why would individuals providing health promotion or health care at the community level be any different? We have now entered the age where this principle is recognized by both advocates and technical authorities,^1^ although practice struggles to follow the principle.^9^^,^^10^ Given the scale of CHW programs required to achieve their potential, appropriate compensation has a nonnegligible cost, even if the investment is cost-effective.^9^^,^^11^ Does this mean, however, that volunteerism should be retired completely once governments and programs have budgeted for enough CHW staff costs? Probably not.

Volunteerism exists in community work, in organizing global health conferences, as a learning opportunity for young (and not-so-young) professionals through internships, for the arts, and for 1,001 community life activities, fairs, political and faith-based activities, not to mention writing commentaries. It is called “volunteerism” precisely because it is an exception to the established principle that “workers are worthy of their hire.” While abuses of the past are rightly recognized, volunteerism has its own benefits to volunteers. People who volunteer report better health and greater happiness than people who do not, a relationship that is not driven by socioeconomic differences between volunteers and nonvolunteers.^12^ Volunteerism has health benefits including reduced hypertension and increased psychological well-being.^13^

Additionally, if we consider the role of social support and social capital in community health,^14^ the potential of broad community engagement processes, the very scale of PHC efforts trying to reach “the last child” and neglected communities, and the strength of people coming together, it would be foolish, counterproductive, and even patronizing to toss aside volunteerism as a resource. In the end, what global health “influencers” should stand up against is the abuse of volunteers when long hours (e.g., more than 4 hours per week) are required of people with limited agency and who are faced with a precarious livelihood. In the aforementioned supplement, Colvin et al.^7^ remind us of the WHO recommendation on compensation, namely of commensurability with the job demands and of explicit formulation through written agreements. While the CHW-community health volunteer (CHV) “dual model” requires clearly differentiating the management of a professionalized workforce (paid CHWs) from the engagement of volunteers—and there are fears that dual models might still be exploitative^16^—it seems to be a promising approach for efficiency and scale.^9^^,^^15^ Volunteerism is a force in societies, and we have examples of health sector interventions that have learned to tap into communities' own organizing potential.^15^^,^^17^^–^^21^

If we consider the role of social support and social capital in community health, the very scale of PHC efforts trying to reach “the last child,” and the power of people coming together, it would be counterproductive to toss aside volunteerism as a resource.

Beyond payments, there are multiple ways to support workers' intrinsic motivation and performance. It is reassuring and perhaps humbling that what we are learning about motivation of CHWs aligns with prior lessons from the management and psychosocial sciences. Examples can be useful. The seminal work of Richard Ryan and Edward Deci on self-determination theory posits that individuals have 3 basic psychological needs: autonomy (or self-determination), relatedness (or belongingness), and competence.^22^^,^^23^ Accordingly, the satisfaction of these needs leads to intrinsic motivation. In another model, Josh Epstein's Agent_Zero agent-based model,^24^ an individual's (or “agent's”) “disposition to act”* is based on 3 immediate drivers: cognitive or volitional, emotional, and social (a form of “peer contagion”). The similarities with the drivers of motivation for other behaviors—such as vaccine uptake—are notable.^25^^,^^26^ In previous unpublished work, Save the Children used this model to review 95 publications from the literature on CHWs and human resources for health, business and management, and psychology. This review identified a nonexhaustive list of more than 120 factors of motivation (sometimes external, sometimes internal, sometimes overlapping), which are associated with Epstein's 3 drivers of motivation.^†^ Cognitive factors include the type of professional goals, difficulty of tasks, sense of efficacy and skills, professional opportunities, rewards and compensation, workload, etc. Emotional factors include recognition and support from members of the community, feeling valued, respected, and supported by health system supervisors, pride, and sense of purpose. These positive emotional factors have negative corollaries when absent, such as anxiety and feeling overwhelmed or isolated. A large behavior change program in Mozambique^21^ found that the respect gained from other people in Care Group Volunteers' social network—and seeing the results of the project—were key motivators for volunteers (in addition to cognitive factors, such as skills development). All surveyed volunteers reported that they were more respected by other women in their community because of their participation as a volunteer. They reported being more respected by community leaders (64%), their husbands (61%), their parents (48%), and health facility staff (25%). Global lessons confirm the findings of this study.^27^^,^^28^

The different processes, which can influence cognitive and emotional drivers are known, and their effects on one or the other are sometimes difficult to tease out. Quality supportive supervision, for example, can boost both cognitive factors (skills and competencies) and emotional ones (feeling valued and supported). Other processes that affect motivation include training, equipping, providing compensation, a title, a career path, but also symbolic and practical things such as providing a uniform, a backpack, or a public recognition ceremony.

Coworkers can contribute to both drivers, but Epstein's model acknowledges a direct influence of peers' motivation on an agent's own motivation. This “peer contagion” is a phenomenon known to both team managers and social network researchers. Peer contagion happens when social contacts with other CHWs reinforce positive or negative overall motivation. This can be through geographic proximity, formal group meetings, informal peer support, competition, learning and sharing experiences, and increasingly through digital communication. In simple English, the feelings, motivation, and energy of those close to or like us influence our feelings, motivation, and energy in the workplace, both positively and negatively, and this is also true for CHWs.^29^ Effective program management must be cognizant of these factors and influence positively the cognitive and emotional motivation of CHWs, as well as their group dynamics. The link between motivation and performance is however dependent on context and systems' conditions, which enable or disable performance.^30^ The proverbial “no commodities, no program” may be an illustration of how heavily these constraints can undermine the best programmatic efforts and intrinsic motivation.

The recent work by Colvin et al.^7^ reviews a considerable body of work on the topic but focuses on the incentives provided to CHWs, which are organized under 4 categories: financial, nonfinancial, health system, and community level. This leads us to rapidly consider the systems giving life to CHW programs, before indulging in the risky business of predictions.

## CHW PROGRAMS, FROM PROJECTS TO SYSTEMS

The first question for us, donors, national leaders, program practitioners, and researchers, is why we would think that the motivation of CHWs should be any different from that of other mortals in their respective workplaces? The PHC case for the value of CHWs for delivery of positive health outcomes—as part of comprehensive and coherent strategies—has been made and made repeatedly over half a century.^31^ CHW programs can work and deliver, this we know. What we—starting with national, state, and nonstate leaders—need to get better at is to shift from epidemiological and disease control mindsets (“what works?”) to organizational and management paradigms (“how can it work better?”) and properly address all incumbent duties of leadership, organization, and management of programs. This demands that we properly understand the systems in which these programs need to be anchored and that we manage accordingly. At a macro/policy level, this fits into a more comprehensive view of health systems, hopefully now broader than the useful but dated “building blocks,”^32^ notably because we need to work with community-level systems and their levers.^27^ Country and program leaders need to operate from a community-inclusive system for health perspective.^15^ At the micro, subnational, and implementation level, the CHW logic model^33^ (Figure) presents a compelling description of the dual anchoring of CHW programs in both institutional (a still traditional “building blocks” view of health systems) and social (communities) realities and offers a useful systems map reflected in other narratives and tools.^27^^,^^34^ The sum of these elements represents a health system as initially intended, differences in models reflecting differences in the central analytical perspective, not in the overall reality.^15^^,^^28^^,^^32^^–^^34^

**FIGURE:**
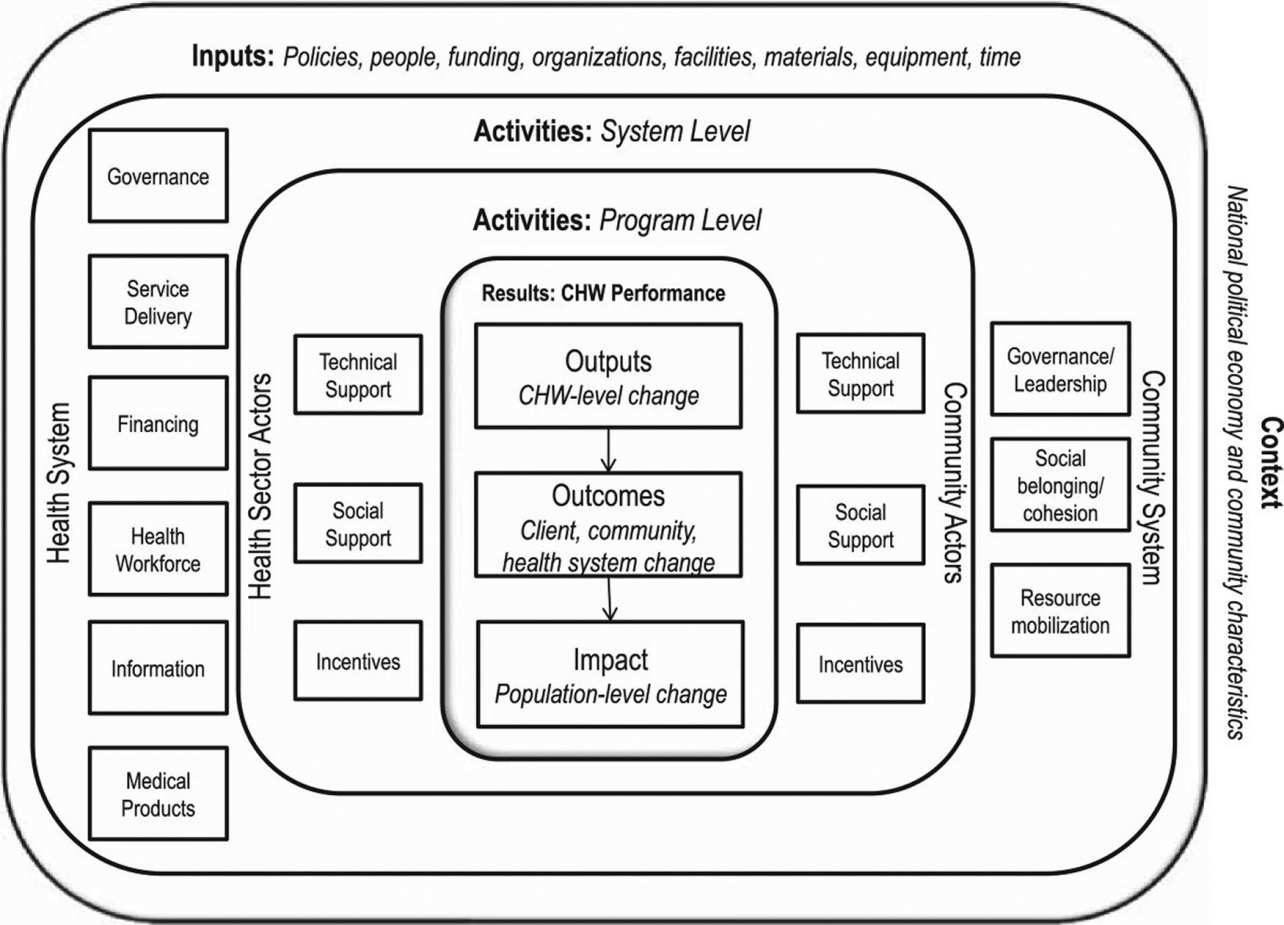
The Community Health Worker Generic Model Source: Naimoli et al.^33^

At this point, we need to shift from asking “what works?” in CHW programs, to “how can they work better?” and “how can we guide and support CHWs?”

While aware of the variously attributed statement, “predictions are difficult, especially about the future,” we would like to conclude with 4 overarching predictions about where these management and systems considerations may take us in the future.

### 1. National CHW Programs—Whether Implemented by the State or Nonstate Partners—Will Be a Growing Opportunity to Viably Strengthen National Health Systems

At times, there is an unspoken sense that when health norms and outcomes improve or when GDP per capita gets higher, we will somehow end up with our health needs taken care of by hospitals, clinics, health facilities, and doctors, and finally be free of CHW programs, which we stigmatize as a lower-income country, nongovernmental organization (NGO)-driven “thing.” This is perhaps more of a blind spot than as an explicit statement by thought leaders in our field (see for example a recent call to rethink health systems, which entirely skips the necessary investments for community health^35^). But CHW programs are not an artifact of external development assistance; they are a central and adaptable part of effective and viable systems for health, regardless of the income status of a country. High-income countries may have been able to project a mistaken facility-centric vision of health but only for a time. Throughout HICs, we see breaches in the connection of health systems to communities possibly due to this blind spot, for example, through vaccine hesitancy and refusal and the huge burden that unhealthy lifestyles have on health status in HICs. In LMICs, there was a major learning moment at the time of Ebola, and we have now a reckoning about the necessity of community health and CHWs in this time of novel coronavirus disease (COVID-19).^36^^,^^37^ Interestingly, this moment is shared by LMICs and HICs. The literature on CHWs in HICs is slowly growing from addressing COVID-19, access to health care, and noncommunicable diseases, among others.^‡^^38^^–^^46^ Of course, epidemiological, economical, and demographic conditions will ultimately drive CHW programs in different directions, for example, toward noncommunicable diseases or toward universal health coverage-related access and social issues.

The need for health-focused CHWs in LMICs is with us to stay, even for some curative services,^47^ but time will tell whether we will see an evolution toward the social side of the health and social services spectrum over time.^48^ Roles cannot be expected to be static in an ever-changing environment, but what should be expected from good governance and management is clarity of roles, focus on achievable (“do-able”) missions, fitness to the needs and structures of primary health care, compensation, and proactive and diligent management.

### 2. Partnership for Effective PHC and CHW Programs Will Be a Keyword of Our Future Success

While the governance of CHW programs needs to fit under 1 national health strategy, this strategy will be able to choose from a plurality of possible state or nonstate partners as program managers. We now have plenty of evidence for both nationally run CHW programs^45^ and for programs run by NGOs and even private hospitals seeking to expand their PHC activities. The stigmatizing of “parallel” NGO programs, if perhaps occasionally deserved, is probably also a reflection of governance gaps. It seems to disappear where governments are deliberate in advancing community health and in creating constructive partnerships for public goods with civil society.^§^ NGOs have often provided a learning platform and testing ground for innovations,^52^ and they are a somewhat irreplaceable force for mobilization and organization as soon as new threats emerge, from HIV to Ebola to COVID-19. In Maryland, United States, the Baltimore Health Corps was born under the impetus from faith-based organizations, social justice groups, Johns Hopkins University, and the city government. Partnership will endure as a keyword for future PHC programs and will be key to the performance and adaptability of CHW programs.

Dual models of paid CHWs and volunteers will need to be effectively and ethically managed, with proper differentiation of their status, roles, and motivations. It may be unrealistic to expect governmental structures to manage with the required agility the mobilization of volunteers, which, by definition, will come and go. Partnership of the health sector with local government and with civil society organizations has already shown strong potential and will continue to be needed. We need to move beyond all-or-nothing debates, whether debates on paid CHWs and volunteer programs or on the complementary role of the state and civil society, especially since both have legitimacy and need of one another. The health of the public depends on both. Ultimately, nations—inclusive of citizens, civil society, private entities, and the state—need to choose the governance and PHC architecture that suits them to face evolving challenges and to fine-tune arrangements in an ongoing manner. Local adaptive management, negotiation, and learning within pluralistic health systems need to take over global (well-meaning) one-size-fits-all mandates.

Dual models of paid CHWs and volunteers will need to be effectively and ethically managed, with proper differentiation of their status, roles, and motivations.

### 3. The Social-Versus-Institutional Anchoring of CHW Programs Will Be an Enduring Challenge for Their Performance and Sustainability

We will continue to evolve our programs and will need to differentiate between CHWs and CHVs. This will require adaptive contextual processes. CHWs were born out of a need to anchor health promotion within communities. Selection of CHWs by their communities to ensure both their integration in the social landscape and community ownership has been an essential feature of CHW programs. This remains the case, but the dual anchoring of CHWs is progressively going to shift from communities first to the “health system” as CHWs are expected to provide more and more professional work and hours with hopefully more and more appropriate compensation. This professionalization comes with a shift in accountability (from communities to health structures), the appeal of career paths, and the geographic mobility this affords. There is also the temptation from managers in frontline facilities to pull CHWs away from community work to help with the limited staffing of their facilities. This pull may be attractive to some CHWs themselves, who may find time in facilities to be perhaps easier, certainly providing more shade in hot climates, and more social opportunities than long treks and walks to reach the last mile. Finally—as some of our co-authors have already observed—professional CHWs are becoming more educated, and this will out-select some community resource persons who would have become CHWs under a previous model. All in all, it does not require a behavioral economist to acknowledge that programs with professionalized CHWs will have the unavoidable tendency of moving CHWs from a social (community) to an institutional (facility) anchoring. Again, dual models with both volunteers (CHVs) and professional CHWs may be a possible solution. This complexity will need careful management.

At this point, the line between the paid CHWs and CHVs often remains blurred. After all, the WHO recommendations are only 4 years old, and it takes time to evolve out of old program habits. Furthermore, policy decisions can have significant budgetary implications, and large program shifts are subject to “path dependency”: once a direction has been taken, it may be challenging to reverse. Governments are consequently cautious about budgets, and budgets are dependent on habits, which are difficult to change. Many countries are still dealing with different types of CHWs, providing a range of different services, under a range of varied compensation and financial incentive schemes, delivered by different managing institutions. Contextual scenario mapping, planning, problem solving, optimization through operations research, ongoing learning, and adaptive management will be key in guiding these programs toward coherence, performance, and sustainability.

### 4. *Effective* National Programs Will Be Led by Managers Who Expend Energy to Uncover and Optimize the Drivers of Motivation of Their CHWs at the Intersection of Their Institutional and Social Anchoring

Returning to the motivation of CHWs, we can now recognize that motivation can only be built on top of policy, program design, social anchoring, and institutional management platforms. While service organizations operating at scale can make short-term gains by pressuring their human resources, the long-term health of organizations (not to mention ethics) rests on a competent and motivated workforce. Health systems, or rather community-inclusive systems for health, are no different.

The job of CHW program managers—along with country policy makers, civil society actors, and researchers—will be to identify (and adjust at appropriate times) what combination of factors under their influence can advance the internal cognitive, emotional, and peer-influence factors of CHW motivation. This demands proactive steps in management. Our research models must consequently evolve to address more specific questions. CHW program managers will have to combine lessons from psychosocial research with management science, operations research, and qualitative studies to improve the effectiveness of their programs step by step, through a motivated workforce.

CHW program managers will need to identify (and adjust at appropriate times) what combination of factors under their influence can advance CHW motivation.

Interestingly, Roy et al. show us what it takes to turn a “paper workforce” into motivated human capital, starting with timely and quality research.^4^ Colvin et al. usefully seek to translate 4 types of CHW incentives from their review (see above) into a dozen or so policy and management “prompts,” which will deserve consideration context-by-context.^7^

Our research and evaluation need to evolve from one-off testing of project interventions (a new external incentive, a new process creating a new cadre for a new task) to the consideration of the effective implementation of programs through the dynamic challenges of time. This requires a view of programs as ongoing systems, which need to ethically support the intrinsic motivation of CHWs—cognitive, emotional, and social.

Finally, context not only matters, but it centrally matters. The world is increasingly urbanized, and the health benefits of urbanization are unequally distributed, with peri-urban, informal settlements, and megacities' poverty bringing a new scale of challenges. CHWs have operated in both rural and urban contexts, but in LMICs, rural has dominated. More research and learning will be needed in urban contexts.

## CONCLUSION

Global patterns and lessons about CHW programs will continue to emerge, but it is local, national, and contextual applied research to hear the voices of CHWs and inform proactive management that will move us forward. National leaders must step forward, in LMICs and HICs, to internalize and vocally acknowledge their dependence on effective community health to achieve and sustain the ambitions of “health for all.” We need to accelerate the development of viable community health platforms now (or preferably 20 years ago^53^). Governments need to see that CHWs, CHVs, communities, and development partners are central to this agenda. But we have been warned: while cost-effective, CHW programs are not cheap, and they will fail again, unless they are managed professionally^54^ as part of viable systems for health, balancing institutional with social anchoring.^15^^,^^27^^,^^28^ The bottom line is that programs need to invest in their human resources, understand them, and support them. What workforce ever performs well when management does not **invest** in its ownership and its motivation in the mission of the organization?

There is nothing new under the sun. And yet…

## Footnotes

* “Motivation” can be a confusing word in development as it refers to both an internal disposition and to means for promoting the individual disposition, which effectually leads to performance if contextual conditions allow it. In this article and to keep it simple, we will use “motivation” to mean the internal propensity of an agent to take action, the “disposition to act” (in the Epstein model).

† Evaluators of the realist persuasion may recognize these as “mechanisms” of the Context-Mechanism-Outcomes model.

‡ See example in Switzerland with the Agents de Santé (https://agentsante.ch/), in Baltimore, Maryland with the Baltimore Health Corps (https://www.baltimorecorps.org/baltimore-health-corps).

§ A full review of this issue goes beyond the scope of this article, but Bangladesh, Ethiopia,^49^ Ghana,^50^ Nepal, and Rwanda^51^ come to mind.

## Notes

**Peer Reviewed**
